# Twenty-first century hydroclimate: A continually changing baseline, with more frequent extremes

**DOI:** 10.1073/pnas.2108124119

**Published:** 2022-03-14

**Authors:** Samantha Stevenson, Sloan Coats, Danielle Touma, Julia Cole, Flavio Lehner, John Fasullo, Bette Otto-Bliesner

**Affiliations:** ^a^Bren School of Environmental Science and Management, University of California, Santa Barbara, CA 93106;; ^b^Department of Earth Sciences, University of Hawaii at Manoa, Honolulu, HI 96822;; ^c^Department of Earth and Environmental Sciences, University of Michigan, Ann Arbor, MI 48109;; ^d^Department of Earth and Atmospheric Sciences, Cornell University, Ithaca, NY 14850;; ^e^Climate and Global Dynamics Laboratory, National Center for Atmospheric Research, Boulder, CO 80303;; ^f^Institute for Atmospheric and Climate Science, ETH Zurich, 8092 Zurich, Switzerland

**Keywords:** drought, climate change, hydroclimate, extreme events, large ensembles

## Abstract

Variability in hydroclimate impacts natural and human systems worldwide. In particular, both decadal variability and extreme precipitation events have substantial effects and are anticipated to be strongly influenced by climate change. From a practical perspective, these impacts will be felt relative to the continuously evolving background climate. Removing the underlying forced trend is therefore necessary to assess the relative impacts, but to date, the small size of most climate model ensembles has made it difficult to do this. Here we use an archive of large ensembles run under a high-emissions scenario to determine how decadal “megadrought” and “megapluvial” events—and shorter-term precipitation extremes—will vary relative to that changing baseline. When the trend is retained, mean state changes dominate: In fact, soil moisture changes are so large in some regions that conditions that would be considered a megadrought or pluvial event today are projected to become average. Time-of-emergence calculations suggest that in some regions including Europe and western North America, this shift may have already taken place and could be imminent elsewhere: Emergence of drought/pluvial conditions occurs over 61% of the global land surface (excluding Antarctica) by 2080. Relative to the changing baseline, megadrought/megapluvial risk either will not change or is slightly reduced. However, the increased frequency and intensity of both extreme wet and dry precipitation events will likely present adaptation challenges beyond anything currently experienced. In many regions, resilience against future hazards will require adapting to an ever-changing “normal,” characterized by unprecedented aridification/wetting punctuated by more severe extremes.

## Significance

Twenty-first century trends in hydroclimate are so large that future average conditions will, in most cases, fall into the range of what we would today consider extreme drought or pluvial states. Using large climate model ensembles, we remove the background trend and find that the risk of droughts and pluvials relative to that (changing) baseline is fairly similar to the 20th century risk. By continually adapting to long-term background changes, these risks could therefore perhaps be minimized. However, increases in the frequency of extremely wet and dry years are still present even after removing the trend, indicating that sustainably managing hydroclimate-driven risks in a warmer world will face increasingly difficult challenges.

Around the world, hydroclimate variations have severe impacts across human, ecological, and economic sectors. These variations occur across multiple interacting temporal scales; for instance, California has been in drought conditions for the majority of the millennium to date (1), yet experiences strong intra- and interannual precipitation variability that has significant impacts on regional water availability and disaster preparedness (2). Successfully managing the impacts of future extreme events thus depends critically on understanding hydroclimate variability across timescales.

Internal variability in the climate system has a major impact on hydroclimate. These variations arise stochastically (3) due to many processes in the coupled Earth system. Their magnitudes depend on the timescale and variable of interest, but are comparable to the magnitude of anthropogenic climate change on decadal timescales (4). The true extent of internal hydroclimate variability cannot be estimated from the single available realization of the observational record. Using paleoclimate information to extend the observational baseline provides improved statistics, but presents additional challenges of interpretation (5). Although also imperfect representations of reality, large ensembles of climate model simulations can provide more comprehensive estimates of the possible range of internal climate variations. Single-model initial-condition large ensembles (SMILEs) are particularly well suited to assessing these uncertainties. By perturbing the initial climate state, divergent climate trajectories can be created that follow the same underlying physics and therefore sample from the same distribution of internal climate variability. Since different climate models simulate different magnitudes of internal variability (6), however, it is necessary to consider multiple large ensembles run with different models. This incentivized the creation of the Multi-Model Large Ensemble Archive (MMLEA) (7), an unprecedented compilation of SMILEs run at various modeling centers.

In addition to internal variability, climate change is expected to drive large background trends in hydroclimate. These trends may be toward either aridification or increased moisture (8), with regionally complex spatial structure. By the end of the 21st century under high-emissions scenarios, background trends are expected to dominate internal variability in driving regional drought risk (9). However, these effects are highly regionally variable and are influenced by the spatial pattern and hydrological properties of vegetation (10–12), which are often not well represented in climate models. Once again, SMILEs have unique potential to address this issue, since the many available realizations of each model provide robust estimates of the forced background trend, and the analysis of multiple ensembles quantifies the uncertainty driven by intermodel structural differences.

This work applies the MMLEA in an as yet underexplored context, with the aim of understanding changes to low- and high-frequency hydroclimate extremes relative to a continuously changing forced background trend. Our analysis has critical implications for resource management: For instance, previous work has shown that the risk of a multidecadal “megadrought” event is expected to exceed 99% by the end of the 21st century in some regions (9, 13). This result begs the question of how one should properly define a drought event, in a situation where the background state is shifting. SMILEs allow us to accurately remove the forced signal and isolate internally driven hydroclimate variability, even in a changing climate.

Removal of the forced background trend also allows us to isolate the effect of changes to high-frequency events. Changes to precipitation extremes are a robust feature of future climate projections (14, 15), owing to changes in the underlying statistical distribution of precipitation (16). As is the case for decadal hydroclimate variability, even if the impacts of forced background trends can be mitigated, changes to higher-frequency extremes could still pose a substantial challenge for water management and disaster preparedness efforts. A statistically robust removal of the forced background trend makes it possible to directly quantify this effect.

Here we apply the MMLEA database to hydroclimate variability, considering changes to hydroclimate variability on two timescales: decadal megadroughts/pluvials and seasonal/interannual precipitation extremes. This approach allows us to ask the following question: If a shift to a drier/wetter background state is occurring in many regions, when do excursions now considered as megadrought/pluvials stop being discrete events and become the norm? Importantly, we are also able to determine how severe the impacts of future extremes will be, relative to the continually evolving background state.

## Mega-Events and the Continuously Shifting Baseline

Data are taken from the Multi-Model Large Ensemble Archive, compiled at the National Center for Atmospheric Research from simulations run at modeling centers around the world (7). All of these ensembles were created using the “high-emissions” Representative Concentration Pathway 8.5 (RCP8.5), and four of these models made the appropriate land surface variables available: the Community Earth System Model version 1 (CESM1), the Geophysical Fluid Dynamics Laboratory Coupled Model version 3 (GFDL-CM3), the Canadian Earth System Model version 2 (CanESM2), and the Commonwealth Scientific and Industrial Research Organisation Mark 3.6 (CSIRO Mk-3.6) (*Materials and Methods*). The choice of the RCP8.5 high-emissions scenario for this study was necessitated by data availability, since all available SMILEs employed this scenario in their experimental design. We note, however, that RCP8.5 is regarded by some as an overestimate of projected future warming (17) and a lower-emissions scenario will project a less extreme set of future changes. Nonetheless, these ensembles represent one of the best available experimental suites to assess the potential range of future hydroclimate changes, and the complete sets of simulations used, including ensemble sizes, are described in *SI Appendix*, Table S1. Megadrought and megapluvial events are defined as in a previous study by Ault et al. (9), using a ±0.5*σ* exceedance threshold in the 15-y running-mean soil moisture. This definition corresponds roughly to values associated with recent historical events: for instance, the 1996 to 2012 “Big Dry” in Australia or the 1930s Dust Bowl drought in the Central Plains of the United States (*SI Appendix*, Fig. S1). We standardize soil moisture data using the corresponding preindustrial control simulation data taken from the Coupled Model Intercomparison Project 5 (CMIP5) archive (*Materials and Methods*).

**Fig. 1.**
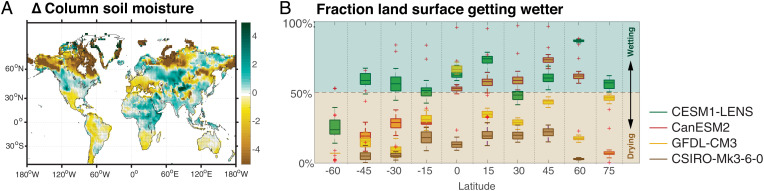
Multiensemble mean changes to conditions in the 21st century (2040 to 2080) relative to the 20th century (1950 to 2005). (*A*) Change in total column soil moisture (*σ*), standardized relative to the SD of monthly soil moisture in the PI control for each ensemble. Stippling indicates locations where the 21st to 20th century difference is not robust, defined as two or fewer of the large ensemble means agreeing on the sign of change. (*B*) Fraction of land surface points experiencing a positive change in total column soil moisture, for 15^∘^ latitude bins. Box width corresponds to interquartile range for each ensemble; line within the box indicates the ensemble median.

The 21st-century hydroclimate changes in most regions under RCP8.5, compared to 1950 to 2005, are robust across models (Fig. 1), with at least three of the four ensemble means agreeing on the sign of soil moisture change across much of the global land surface (*SI Appendix*, Figs. S2 and S3). The pattern of change is broadly consistent with previous analyses of CMIP5- and CMIP6-era simulations (8, 18, 19): More than half of land grid points project drying over the majority of latitudes (Fig. 1*B*), but regional wetting is also projected, particularly for eastern Africa and southeast Asia (Fig. 1*A*). Total column and surface soil moisture results are similar (*SI Appendix*, Figs. S2–S4) and are influenced by both temperature and precipitation changes (*SI Appendix*, Figs. S5 and S6). While there are seasonal differences in column soil moisture trends, they are modest (*SI Appendix*, Fig. S7), consistent with previous work (8).

It is also important to note the potential contributing influence of model biases on intermodel structural uncertainty. For instance, the depths of the soil columns differ across models (*SI Appendix*, Table S1), although we do not find a straightforward relationship between soil depth and soil moisture change. Likewise, despite these differences, there is good agreement across models in projected changes. Intermodel agreement does not, however, necessarily indicate that the changes are realistic, as some biases are common to all models. For instance, model bias in the representation of the precipitation response to forcing has been documented to be significant and common to many models in regions such as the Horn of Africa (20).

**Fig. 2.**
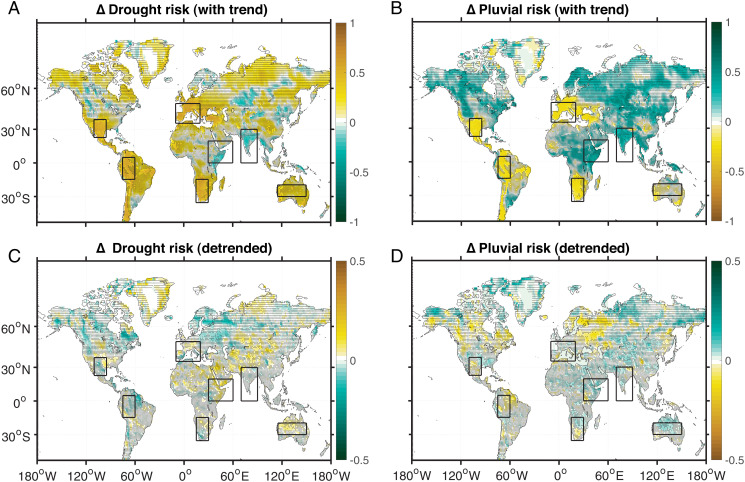
Fractional changes to the risk of mega-events with and without the presence of the background trend. (*A*) Difference in the risk of megadrought between the 21st and 20th centuries, with the background trend included. (*B*) Same as *A*, for megapluvial events. (*C*) Same as *A*, using detrended soil moisture data. (*D*) Same as *C*, for megapluvial events. Total column soil moisture is used for all calculations. Stippling indicates locations where the 21st to 20th century difference is not robust, defined as two or fewer of the large ensemble means agreeing on the sign of change. Boxes superimposed on various panels indicate study regions identified based on the local magnitudes of megadrought/megapluvial risk (see also *SI Appendix*, Table S2 for more detailed definitions).

The risk of megadrought/pluvial occurrence, defined as the proportion of time spent exceeding the relevant threshold, is strongly impacted by the forced background trend in column soil moisture (Fig. 2 *A* and *B* vs. Fig. 1*B*); the same is true for risk estimates derived from surface soil moisture (*SI Appendix*, Fig. S8). Specifically, increases in the 21st vs. 20th century megadrought risk (Fig. 2*A*) are much larger in regions that experience background drying. These differences in megadrought risk are consistent with previous work specific to southwestern North America (9, 21). However, drastic increases in megadrought risk are expected in many other drought-prone regions, with western Europe, southern Africa, Australia, and the Amazon basin being the hardest-hit regions (Fig. 2*A*). Likewise, the risk of megapluvial events generally increases where mean wetting occurs, which is most apparent in India and eastern Africa (Fig. 2*B*). This suggests an increase in risks associated with excessive moisture as well as moisture deficits, highlighting the regionally specific nature of the predicted impacts. Regions associated with relatively large increases in either megadrought or pluvial risk are selected from Fig. 2 *A* and *B* for further analysis (*Materials and Methods* and *SI Appendix*, Table S2).

Other important characteristics of megadrought and pluvial also change dramatically in the 21st century. For instance, both the severity and persistence of events increase substantially (*SI Appendix*, Figs. S9 and S10). An example time series illustrating the influence of the background trend on megadrought properties (*SI Appendix*, Fig. S11) shows that large parts of the globe are effectively experiencing one single megadrought/pluvial event throughout the entire 21st century. In both the megadrought and megapluvial cases, increases in risk accompany enhanced severity and reduced event frequency (*SI Appendix*, Figs. S8–S10). The implications are clear: Although the direction of change varies regionally, background soil moisture trends are fundamentally altering all aspects of megadrought and megapluvial events.

The strong influence of background hydroclimate trends on megadrought/pluvial risk raises a fundamental question regarding how to define these events in a continuously changing climate. If, for some regions, anthropogenic forcing is increasing the probability that each year is drier (or wetter) than the one before, what then should be considered a megadrought/pluvial event as the baseline shifts? For resource managers and other stakeholders, these background hydroclimate trends may be felt as a shift in “normal” conditions (22, 23). This raises complex questions regarding the necessary degree of adaptation: For many applications, interannual variability is the primary concern, but background trends are clearly relevant to long-term management and new infrastructure development. This is apparent, for example, in discussions around flow reductions in the Colorado River Basin in the context of ongoing climate change (24) and in the feasibility of continuing water-intensive agriculture in drought-prone regions. In many such cases, the relevant question when considering the overall impact of climate change is not “How common will extreme drought/pluvial periods be in the future?” but rather “When do we recognize a drought/pluvial period as a shift in what is considered normal (and start managing accordingly)?”

To address the question of the changing background state, we perform time-of-emergence calculations to determine when a high-emissions scenario predicts normal conditions will meet or exceed the threshold for a present-day megadrought/pluvial. The time of emergence is defined as the year in which the ensemble mean exceeds the relevant threshold and does not return. The present approach is similar to standard time-of-emergence procedures (25–27), but rather than using a signal-to-noise metric based on the magnitude of natural climate variability, here we use 0.5*σ* above/below the reference period average as the emergence threshold, this being the threshold used in Fig. 2. We assume that emergence later than 2080 indicates that megadrought/pluvial conditions will not become normal by the end of the 21st century, although this does not necessarily indicate an absence of shifts in the mean state.

**Fig. 3.**
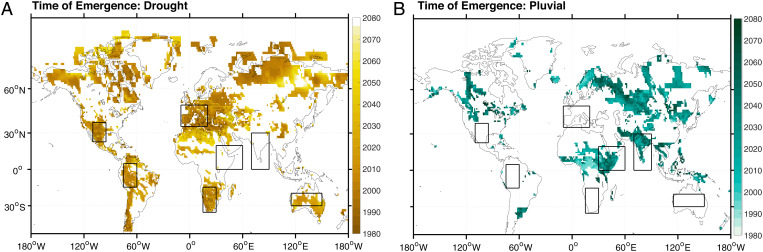
(*A* and *B*) Time of emergence estimates using all large ensembles for megadrought (*A*) and megapluvial (*B*) total column soil moisture thresholds. Soil moisture values have been standardized as in the main text, prior to the determination of time of exceedance for the appropriate threshold (±0.5*σ*). Stippling indicates that emergence occurs in at least three of four ensembles. White regions indicate that the signal does not emerge by 2080, in the RCP8.5 scenario employed by the SMILEs analyzed here. Lower-emissions scenarios would likely result in later estimates of emergence time.

The time of emergence is earlier in regions with stronger background trends: In the southern United States/northern Mexico, the Amazon, the majority of Europe, and southern Africa, the large ensemble projections indicate that megadroughts become normal in the early 2000s (Fig. 3*C*). This is consistent with work indicating recent regional emergence of megadrought conditions (1). In northern Canada, central Africa, and Australia, megadroughts are projected to become normal only later in the century (2030 to 2050). By contrast, in India and the Middle East, among other regions, megapluvial conditions instead emerge as the new normal (Fig. 3 *A* vs. *B*). The majority of the global land surface experiences the emergence of either megadrought or pluvial conditions: Over 50S to 90N, emergence occurs in 61% of land grid points by 2080. Our results suggest that a significant transition in hydroclimate will occur throughout many countries worldwide, necessitating reassessment of how water resources are allocated and preserved.

The time-of-emergence estimates in Fig. 3 are striking, but there are (sometimes substantial) uncertainties arising from intermodel differences. Megadrought is unlikely to become the new normal by the end of the 21st century in most regions in CanESM2 (*SI Appendix*, Fig. S12*B*), consistent with the wetting trends seen over much of the globe (*SI Appendix*, Fig. S3*B*). However, the other three model ensembles show many common features (*SI Appendix*, Fig. S11 *A*, *C*, and *D*), including early megadrought emergence in the south-central United States/Mexico, Europe, and southern Africa and megapluvial emergence in India. Additionally, time-of-emergence estimates derived using surface soil moisture are more consistent across models (*SI Appendix*, Fig. S13), as was the case for changes in mean state.

## Changing Precipitation Extremes

The intensity and frequency of extreme rainfall events, which are superimposed on the hydroclimate shifts discussed above, have strong implications for managing water storage in reservoir systems during otherwise dry periods and affect flood mitigation strategies. Twenty-first century warming is widely expected to lead to changes in both wet and dry extremes: Increases in the intensity of extreme precipitation are robustly projected by climate models (14, 15), and changes to dry extremes are projected due to land surface aridification and a longer return period for moderate to intense rainfall events (2, 28, 29). However, the precise extent of these increases and their spatial patterns are subject to uncertainty, particularly over land (30).

**Fig. 4.**
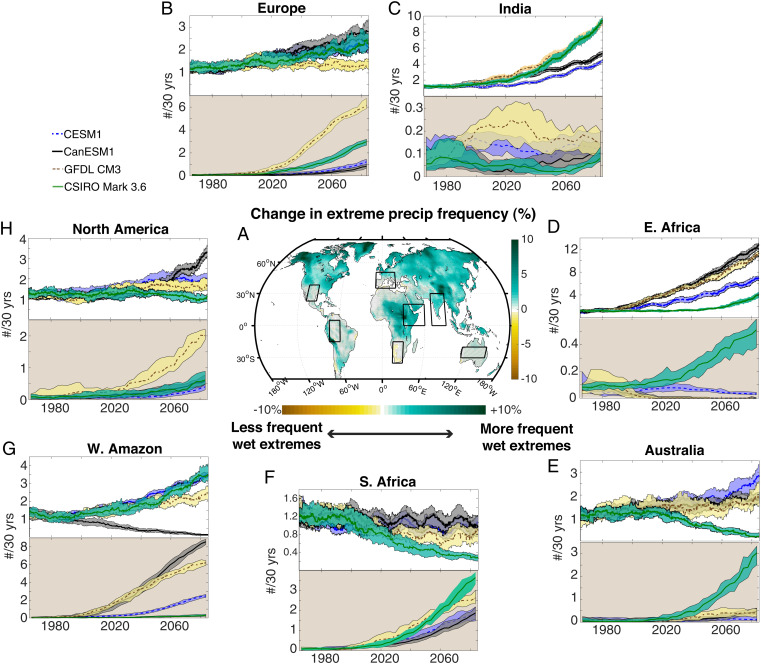
Changes to precipitation extremes in large ensembles. (*A*) Spatial pattern of relative changes to the occurrence frequency of wet extremes. Stippling indicates locations where fewer than three large ensembles agree on the sign of 21st vs. 20th century changes. (*B*–*H*) A 30-y running time series of occurrence of wet (*Upper*) and dry (*Lower*, brown shading) extremes, for the seven study regions delineated in *A*. Solid lines indicate the median for each ensemble, and the shaded envelopes correspond to the ensemble interquartile range.

Here, we examine the consistency of changes to both wet and dry precipitation extremes in the MMLEA (Fig. 3). Definitions for wet and dry extremes are based on previous work on California hydroclimate (2), where wet extremes are considered to operate on seasonal (90-d) timescales and dry extremes on interannual (3-y) timescales. These timescales and thresholds also roughly correspond to impactful extremes observed in other parts of the world (*Materials and Methods*). The spatial pattern of changes to extremes features stronger wet events in the equatorial Pacific and over much of the mid- to high-latitude land surface (Fig. 4*A*), with stronger dry events in the subtropics (*SI Appendix*, Fig. S14).

Examining regional averages, we next find that the frequency of wet extremes increases in most study regions (see Fig. 2 and *SI Appendix*, Table S2 for definition) for the majority of model ensembles (Fig. 4 *B*–*H*). Southern Africa is an exception, with wet extremes becoming less common in all ensembles, consistent with the behavior of CMIP5 models (31). However, extreme dry periods also become more common in the future in some regions. This is particularly apparent for the Europe, North America, southern Africa, and western Amazon regions. In these cases, three or more ensembles project a large (factor of > 2) increase in the frequency of future dry extremes by the end of the century. This is consistent with previous work showing that although extreme precipitation events increase in the future, the intervals between such events become longer (28, 32). In these regions, it will be necessary to prepare for ever-wider swings in hydroclimate from year to year.

These changes in the statistics of precipitation extremes contribute to the soil moisture changes that lead to the emergence of megadrought or megapluvial conditions. Locations where dry extremes increase most substantially are also places where megadrought risk increases most (Fig. 2*A*), suggesting that the diversity of regional projections of dry extremes may affect the long-term risk of megadrought. Likewise, the two regions where megapluvial risk is robustly projected to increase (eastern Africa and India) show the least robust changes to extreme dry events, although these regions may also be among the most strongly affected by model bias in the representation of wet-season precipitation statistics (20). We acknowledge that the relative importance of these effects cannot be quantified here, due to the lack of appropriate land surface model output fields (11) needed to assess the roles that changes to evaporative water demand and vegetative feedbacks play (8, 11, 33). However, these results nonetheless suggest the possibility for shifting extreme precipitation statistics to affect long-term hydroclimate behavior even when mean precipitation changes are insignificant (e.g., Australia, western United States; *SI Appendix*, Fig. S6).

## Impacts Relative to the Changing Baseline

### Mega-Events.

In regions where emergence of permanent megadrought/pluvial conditions occurs in the early 21st (or late 20th) century, it becomes relevant to consider not only the absolute magnitudes of extremes, but also variations around that changing baseline. This is a unique strength of the present SMILE toolkit, where removal of the background hydroclimate trend gives a robust estimate of the magnitude of megadrought/pluvial events—relative to the changing baseline. This is done by subtracting the time-varying ensemble mean, which is considered the most accurate estimate of the time-varying forced response (6, 34), from each ensemble member. Unsurprisingly, the resulting changes in the risk of megadrought/pluvial conditions (*SI Appendix*, Fig. S8) are much smaller than estimates that include the background trend. Globally, the changes range from roughly ± 30% and in most locations are statistically insignificant (Fig. 2 *C* and *D*). Notable exceptions to this rule are the Amazon, parts of southern Africa, and the northern high latitudes of both North America and Eurasia. In these locations, the relative risk of both megadrought and pluvials decreases, even as mean conditions shift.

There is a tendency for both megadrought and pluvial events to become more persistent even after removing the background hydroclimate trend, although the statistical significance of these changes is limited (*SI Appendix*, Figs. S15 and S16). Events also tend to become more frequent; together with the lower overall soil moisture variance in the 21st century (*SI Appendix*, Figs. S17 and S18), this suggests that transitions into and out of weak megadrought/pluvial conditions become more common. The mechanisms for reductions in soil moisture variance are unclear, but in the high latitudes likely relate to an increased conversion of snow to rain (35), which could decrease soil moisture memory and associated interannual variability. In the Amazon, more complex vegetative processes may be playing a role, and the net effect remains unclear; future CO_2_ increases may modify stomatal conductance and therefore reduce plant water use (36, 37), thereby increasing soil moisture. If this is the case, the resulting changes to surface evapotranspiration can affect boundary layer stability, which could in turn affect regional meteorology (38).

### Precipitation Extremes.

**Fig. 5.**
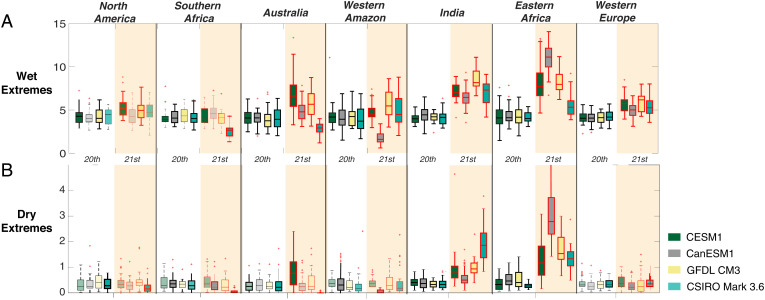
(*A* and *B*) Changes to (*A*) wet and (*B*) dry precipitation extremes (number/century) in large ensembles averaged over the study regions in *SI Appendix*, Table S2, where all precipitation values have been detrended by removal of the time-varying ensemble mean. Boxes are shaded according to the ensemble used; black outlines (no background shading) indicate the historical portion of the relevant simulations, and red outlines (tan background shading) show the 21st century portion. Outlines in boldface type indicate that the difference between the 20th and 21st century portions of the ensembles differs significantly at the 90% level.

Just as adaptation to background trends in soil moisture is possible, adaptation to background changes in the statistical distribution of precipitation could also occur over time. This might take the form of more up to date knowledge of flood risks for evacuation planning or the need to reduce reservoir releases to account for longer intervals between wet years. Precipitation extremes calculated relative to the background trend (Fig. 5) give an indication of the relative impact of these future extremes.

Fig. 5 summarizes wet and dry extreme event frequencies in seven study regions chosen for their relatively large mean-state changes (Fig. 2 and *SI Appendix*, Table S2), for both the 20th and 21st centuries. In every study region considered, the frequency of extreme precipitation still increases in the 21st century, even after removal of the background trend. There are occasional exceptions where an individual ensemble disagrees in a particular region (CSIRO Mark 3.6 in Australia and southern Africa, CanESM2 in the western Amazon) but overall there is strong consensus across regions and ensembles. The consensus for dry periods is much weaker: Only India and eastern Africa project a robust increase in extreme dry event frequency. Taken together, these results describe a future where adaptation to a wider range of wet extremes may be needed, but the range of dry years may change less substantially.

## Discussion and Conclusions

By combining simulations from multiple large ensembles, we have shown that changes to the risk of prolonged drought and pluvial periods are a robust feature across many regions worldwide and that these changes are driven by regionally variable trends in soil moisture, consistent with those documented in previous studies (8, 33). Background soil moisture trends are so large that they dominate or even preclude efforts to identify changes to other properties of mega-events: For instance, large apparent increases in drought/pluvial persistence and severity also take place, and the apparent frequency of events strongly decreases. In other words, background trends are so large that if traditional stationary definitions are used to identify megadrought or pluvial, in those places where the trend emerges, the entire late 21st century is identified as a single large event. Clearly, approaches that account for the continuous changes in mean hydroclimate are needed.

Our time-of-emergence calculations provide a useful conceptual framework for considering the role of a changing background state in driving megadrought/pluvial risk. Although the exact year in which megadrought/pluvial conditions (as defined relative to a 20th century reference period) will permanently emerge above/below the range of historic variability is uncertain, we find that emergence will occur in the next few decades for much of the world under the RCP8.5 high-emissions scenario. Western Europe, the southwestern United States and northern Mexico, southern Africa, and the western Amazon see the earliest emergence of megadrought conditions. Emergence of megapluvials affects less of the global land surface, but does take place in India and eastern Africa. Notably, emergence of either megadrought or pluvial conditions occurs over the majority of the global land surface, for warming consistent with RCP8.5 (roughly 4 ^∘^C in the global mean). Once the megadrought/pluvial threshold is passed, for regions that experience emergence, by definition the risk will become nearly 100% (39) and the entire subsequent period becomes part of a single drought or pluvial event. Therefore, using alternate methods to examine mega-event behavior for these regions becomes necessary.

In our analysis, a shift to a new baseline of megadrought/pluvial conditions has already occurred in many locations. The dearth of long-term soil moisture observations makes it impossible to validate the simulation of soil moisture on multidecadal timescales. However, previous work has demonstrated that the anthropogenic signal in drought is emerging, in many of the same regions used in the present study. For example, increases in drought severity over the Iberian Peninsula have been attributed to increases in temperature-driven atmospheric evaporative demand (40). Similarly, the southwestern United States has experienced prolonged drought since 1999, driven substantially by warming ([1]) and cutting into the region’s main water source, the Colorado River (41, 42). Our results suggest that these trends are unlikely to be reversed under current emissions trajectories, particularly given the strong agreement across multiple large ensembles and for regions where aridification trends are currently being observed. The possibility of mega-event conditions becoming the new baseline in many regions must be taken seriously.

The use of large ensembles allows us to remove the time-varying background trend, as a rough (and optimistic) approximation of the effects of long-term climate adaptation. Relative to this different background, changes to the risk of a mega-event excursion from the 15-y running mean soil moisture are much smaller, and changes in persistence, frequency, and severity of these events are largely insignificant. This might suggest that modifying management practices appropriate for slow, long-term trends might be sufficient. However, when changes to higher-frequency (90 d for wet or 3 y for dry) precipitation extremes are considered, there remain substantial increases in both wet and dry extremes even after removal of the changing background trend. Successful adaptation strategies must therefore account for this additional projected risk of higher-frequency extremes.

In terms of the effects experienced on the ground, it remains important to consider both the factors contributing to background trends in soil moisture and the interaction between trends and internal variability. For instance, the emergence of the temperature signal appears to proceed more rapidly than that of precipitation (*SI Appendix*, Figs. S19 and S20), which in the near term could imply that so-called “hot droughts” (41) might be increasing in frequency. Alternatively, the “delayed” response of precipitation signals could mean that regions where wet conditions are expected to emerge might temporarily experience megadrought due to the near-term temperature trend. In reality, the trajectory of hydroclimate is also substantially influenced by internal variability, such that the presence of a long-term drying trend does not preclude the occurrence of an unusually wet interval (and vice versa).

This work has critical implications for water resource management. Milly et al. (43) famously argued that “stationarity is dead” in water resources: Our results offer a partial autopsy as to how this is occurring for particular regions and variables. Since many regions have or will soon make the transition to a hydroclimatology defined by megadrought/pluvial conditions, water management will need to shift accordingly. Each of the regions we have identified as on the brink of megadrought/pluvial is vulnerable in many aspects: either because of overallocation of managed water resources coupled with rapid population growth, high population density, large biodiversity, and wildfire risk or because of lack of infrastructure for effective drought/flood response. These socioeconomic effects often interact with one another to compound the overall impact of drought events. Perhaps even more concerning, the challenges of responding to future mega-events will be overlain on the increased likelihood of other climate-related disasters (e.g., fire, heat waves, and disease spread) in these already vulnerable locations.

These results are subject to the limitations of model accuracy. Current models are known to have substantial biases in their representation of land surface and precipitation processes (44), as well as in modes of climate variability that can impact megadrought risk (45–48). Models also differ in their soil column depths (*SI Appendix*, Table S1) among many other processes, which may well affect soil moisture trends (*SI Appendix*, Figs. S2 and S3) and time-of-emergence estimates (*SI Appendix*, Figs. S11 and S12). In some regions, the net impact of such biases on the magnitude of background trends is not well known and could lead to our results being either over- or underestimates. Deriving physically based metrics to constrain simulated drought projections will provide a useful way to increase confidence in model projections of future hydroclimate. However, the robustness of our results across multiple large ensembles indicates that future drought and pluvial periods will likely have more severe impacts than anything currently experienced.

## Materials and Methods

The total column soil moisture is used for all megadrought/pluvial computations; this and the surface (0 to 10 cm) values were the only soil moisture variables available across all models, being the standard outputs required by CMIP formatting standards (the “mrso” and “mrsos” variables for total column and surface values, respectively). Total column soil moisture more directly reflects shifts in the overall ecologically available water supply and will thus give a more representative estimate of moisture changes relevant to agricultural/hydrological drought than near-surface values.

### Region Selection.

Seven study regions were chosen for use in regionally averaged diagnostics. These regions were chosen largely on the basis of having a large change in total column soil moisture (Fig. 1*A*); North America, western Europe, southern Africa, and the western Amazon all exhibited a strong drying trend in the 21st century. India and eastern Africa, by contrast, showed a strong wetting trend in the 21st century. Australia was also included despite the lack of significance of soil moisture trends, owing to the large societal relevance and documented history of drought in the region. The geographical boundaries of each study region are listed in *SI Appendix*, Table S2.

### Soil Moisture Standardization.

The *σ* value used for standardization is derived from the preindustrial control simulation for each model, which varies from 300 to over 1,000 y in length, obtained from the CMIP5 database. This is done to avoid spurious increases in variance that arise when the ensemble-member SD over a given reference period is used for normalization (49); in such cases, by construction the reference-period variance is quite uniform, leading to a later apparent increase for time periods outside the reference period. This issue is avoided by the PI control standardization method. However, use of the preindustrial (PI) control mean value for standardizing data leads to a spurious nonzero mean offset. The mean across the set of ensemble-member data covering a fixed reference period is thus used instead (here chosen as 1960 to 1990 to avoid possible influences from the 1991 eruption of Mt. Pinatubo). Megadrought/pluvial risk is defined as the fraction of time spent in megadrought/pluvial conditions and event persistence as the length of the interval between crossings of the 0.5*σ* threshold value. The severity of an event is the average standardized soil moisture anomaly during the period over which soil moisture exceeds the relevant threshold. For “21st century” computations referred to hereafter, the 2040 to 2080 period is adopted, to isolate middle- to end-of-century anthropogenic influences while avoiding end effects associated with the 15-y running mean. When annual or higher-resolution data are considered to assess extremes, all years that contributed to the running mean are included (i.e., if the running mean exceeds the threshold beginning at year 10, years 3 to 17 would be incorporated into the calculation).

### Wet/Dry Precipitation Extremes.

The definitions of wet and dry extremes are loosely based on ref. 2, although the lack of daily precipitation data for all preindustrial control simulations necessitated the use of a percentile-based, rather than a return period-based, threshold. For wet extremes, seasonal (90-d) precipitation accumulations exceeding the 99th percentile over the 1960 to 1990 reference period were selected; this would correspond approximately to an event like the 1976 to 1977 wet season in California or the 2010 to 2011 wet season in Australia. For dry extremes, a 3-y accumulation period is considered since the impacts of rainfall deficits often take longer to manifest. Dry extremes are then defined as periods falling below the first percentile over the reference period. This is roughly analogous to the 2012 to 2016 California drought or the 2018 to 2020 drought in southern Africa. For both the wet and dry cases, percentiles are computed by first concatenating all reference period data from the relevant ensemble and then calculating the moving sum using the appropriate window length. All nonoverlapping windows are selected, and the precipitation threshold is assigned to either the first or the 99th percentile as appropriate.

## Supplementary Material

Supplementary File

## Data Availability

All codes used to perform the present analysis are available in GitHub at https://github.com/samanthastevenson/Stevensonetal2022_PNAS. Previously published data were used for this work (7) and are available at the MMLEA website: https://www.cesm.ucar.edu/projects/community-projects/MMLEA/.
