# Supplementary material for: Twenty-first century hydroclimate: A continually changing baseline, with more frequent extremes
Source: Proc Natl Acad Sci U S A. 2022 Mar 14;119(12):e2108124119. doi: 10.1073/pnas.2108124119 (PMC8944869; doi:10.1073/pnas.2108124119)
Supplement: Supplementary File [file pnas.2108124119.sapp.pdf]

Supplementary Information

21st Century Hydroclimate: A Continually Changing Baseline, With More Frequent Extremes

Samantha Stevenson, Sloan Coats, Danielle Touma, Julia Cole, Flavio Lehner, John Fasullo, & Bette Otto-Bliesner

Correspondence to sstevenson@ucsb.edu

Structure and Content of the Supplementary Information

Table S1: Models and ensemble sizes used for the present analysis

Table S2: Lat/lon limits for regions used in regionally-averaged analyses

Figure S1: Observationally derived PDSI and precipitation time series for study regions

Figure S2: 21st - 20th century differences in surface soil moisture for individual model ensembles

Figure S3: 21st - 20th century differences in column soil moisture for individual model ensembles

Figure S4: 21st - 20th century differences in ensemble-mean surface soil moisture and latitudinal dependence of % grid points experiencing wetting vs. drying

Figure S5: 21st - 20th century differences in surface air temperature for individual model ensembles

Figure S6: 21st - 20th century differences in precipitation for individual model ensembles

Figure S7: Multi-ensemble mean 21st - 20th century differences in column soil moisture, computed for the MAM and SON seasons

Figure S8 : Time of emergence and drought risk change maps derived using surface soil moisture

Figure S9: Changes to the persistence, severity, and frequency of megadrought/pluvial events, derived using column soil moisture

Figure S10: Same as Figure S9, for surface soil moisture

Figure S11: Example time series of SW US/Mexico regional soil moisture with and without detrending, for various model ensembles

Figure S12: Time of emergence maps for megadrought/pluvial events, for each individual model ensemble derived using column soil moisture

Figure S13: Same as Figure S12, using surface soil moisture

Figure S14: Multi-ensemble mean changes to the occurrence frequency of dry extremes in the 21st century relative to the 20th

Figure S15: Changes to the persistence, severity, and frequency of detrended megadrought/pluvial events, derived using column soil moisture

Figure S16: Same as Figure S15, for surface soil moisture

Figure S17: Variance of surface soil moisture in the pre-industrial control simulations run with each model

Figure S18: Same as Figure S17, for column soil moisture

Figure S20: Time-of-emergence calculations for surface temperature

Figure S21: : Time-of-emergence calculations for precipitation

Table S1: Model ensemble sizes used in the analysis, along with the total depths of soil columns and associated references for each.

Ensemble name	Scenario	# Members	Soil depth (m)	Ref. for soil depth
CESM1 Large Ensemble	RCP8.5	30	3.8*	*Lawrence et al.* (2011)
GFDL CM3 Large Ensemble	RCP8.5	30	10**	*Milly et al.* (2014)
CSIRO Mk3.6 Large Ensemble	RCP8.5	30	2.8***	*Gordon et al.* (2002)
CanESM2 Large Ensemble	RCP8.5	50	3.75	*Verseghy* (1991)

*In CLM4, the land surface component of CESM1, the total 'soil' depth is 42m, but all layers below 3.8m are thermal slabs which are not considered hydrologically active.

**This is the 'soil-bedrock domain depth' in LM3.0 (*Milly et al.*, 2014).

***The soil depths in the land surface model remained unchanged between CSIRO Mk3.0 and 3.6 (*Gordon et al.*, 2010; *Jeffrey et al.*, 2013).

Table S2: Regions of interest chosen for regionally-averaged diagnostics, with abbreviations used in Figure 1 in the main text.

**Region name**	**Abbr.**	**Lat**	**Lon**
North America	NAM	23-38°N	110-95°W
Southern Africa	SAFR	15-35°S	15-30°E
Australia	AUS	20-30°S	115-150°E
Western Amazon	WAMA	15°S-5°SN	60-75°W
India	INDIA	0-30°N	70-90°E
East Africa	EAFR	0-20°N	30-60°E
Western Europe	WEU	35-50°N	10°W-20°E

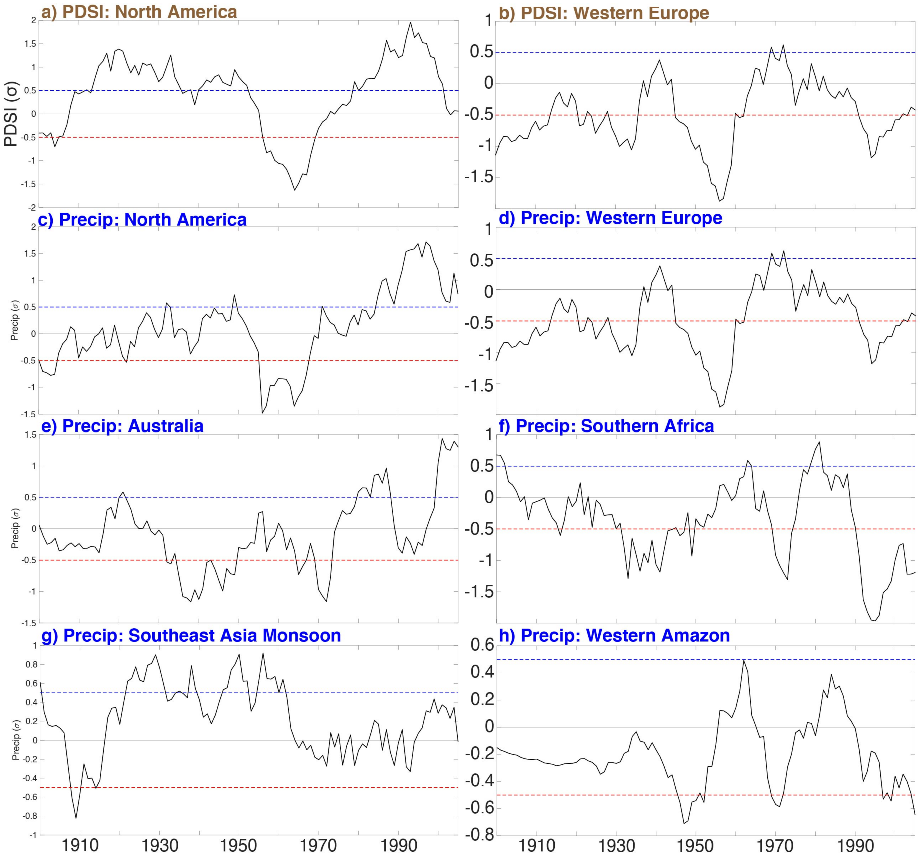

Figure S1: Megadrought/pluvial events in historical observations. a) PDSI over the 'North America' region, from the North American Drought Atlas. b) PDSI over the Western Europe region, from the Old World Drought Atlas. c)-h) Precip over all study regions, taken from GPCC. All data has been standardized to the 1960-1990 period. The 0.5σ threshold for 'mega' events is shown as the red and blue dashed lines.

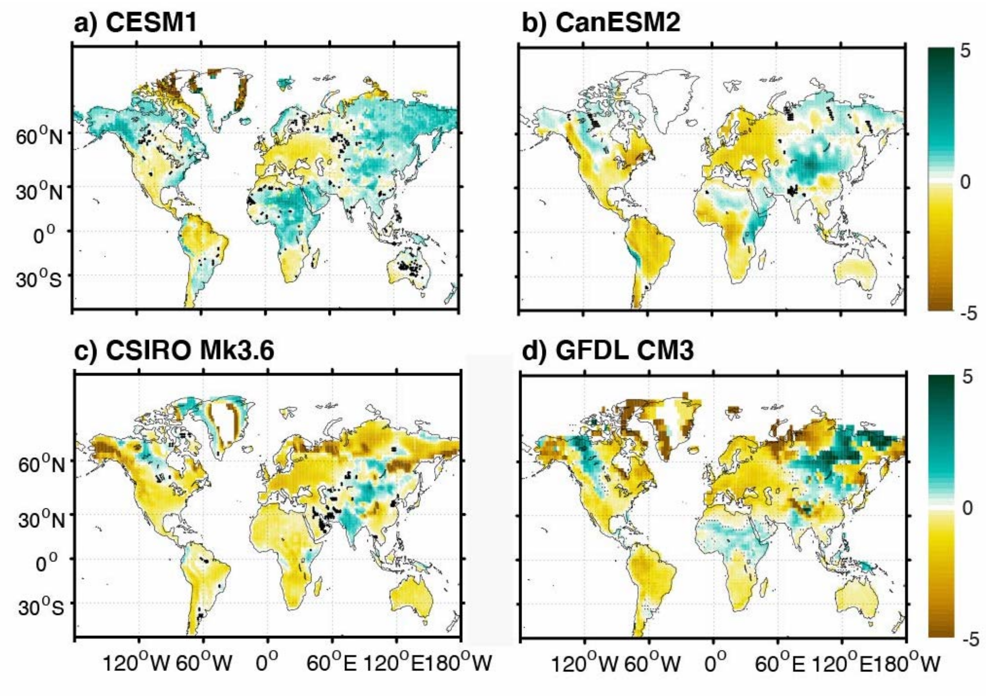

Figure S2: Surface soil moisture difference (σ): 21st c - 20th c. a) CESM1, b) CanESM2, c) CSIRO Mk3.6, and d) GFDL CM3. Stippling indicates that the 21st and 20th centuries are statistically indistinguishable at the 90% level using a Wilcoxon rank-sum test.

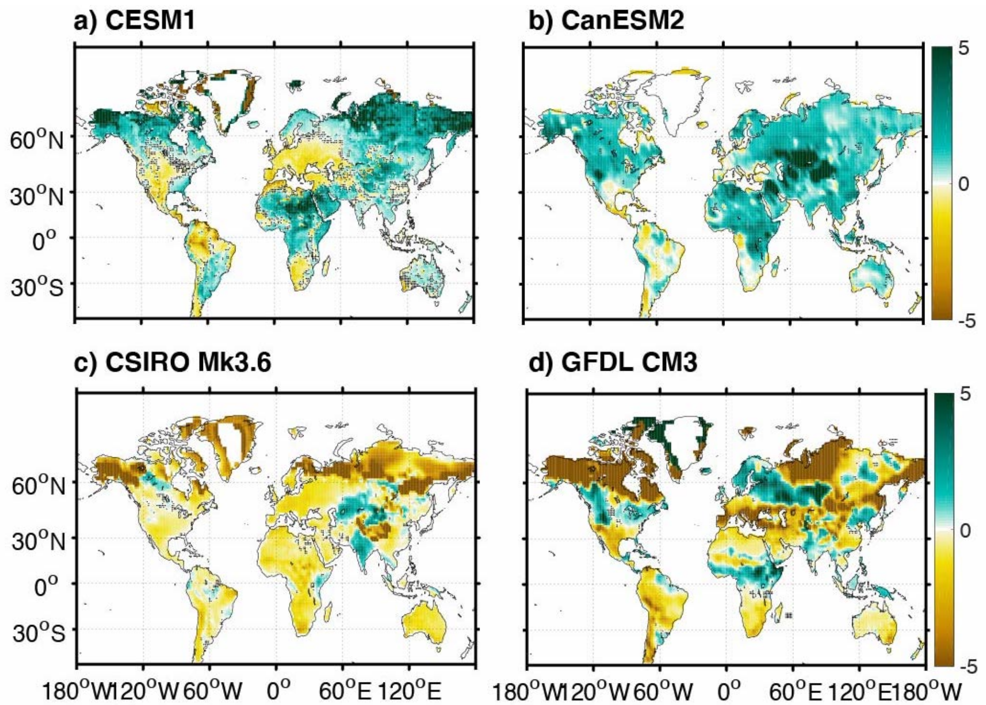

Figure S3: Column soil moisture difference (σ): 21st c - 20th c. a) CESM1, b) CanESM2, c) CSIRO Mk3.6, and d) GFDL CM3. Stippling indicates that the 21st and 20th centuries are statistically indistinguishable at the 90% level using a Wilcoxon rank-sum test.

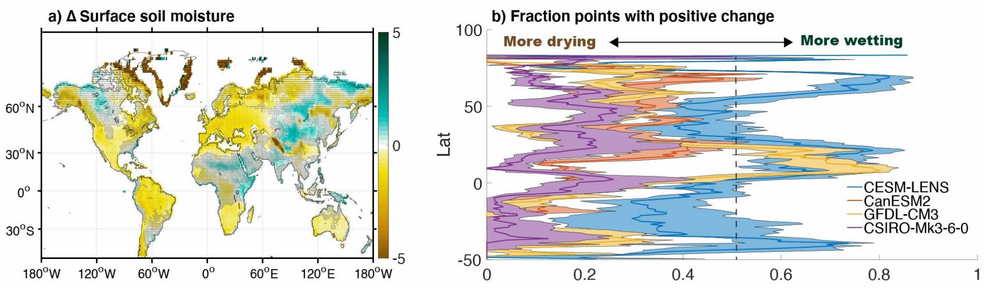

Figure S4: Multi-ensemble mean changes to conditions in the 21st century (2040-2080) relative to the 20th (1950-2005). a) Change in surface soil moisture (σ), standardized relative to the PI control for each ensemble. Stippling indicates locations where the 21st - 20th century difference is not robust, defined as 2 or fewer of the large ensemble means agreeing on the sign of change. b) Fraction of land surface points experiencing a positive change in total column soil moisture, as a function of latitude. Solid lines indicate ensemble median, envelope the interquartile range for each ensemble.

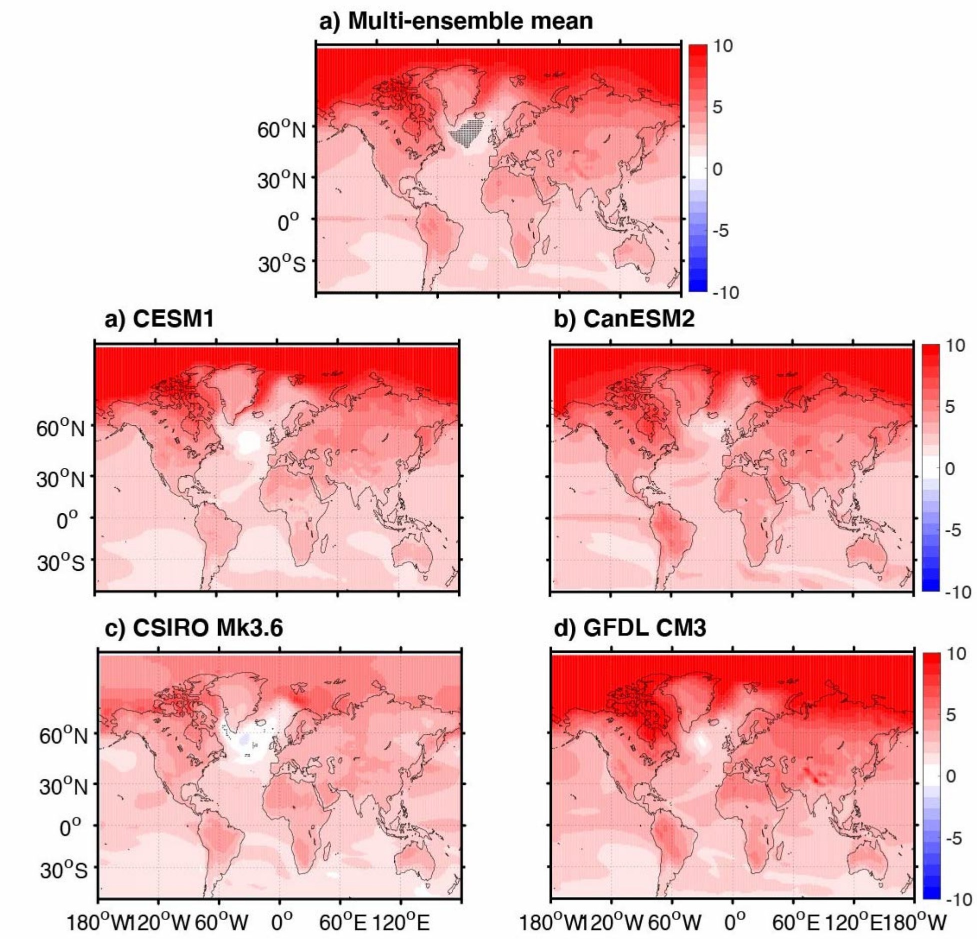

Figure S5: Surface temperature difference (°C): 21st c - 20th c. a) CESM1, b) CanESM2, c) CSIRO Mk3.6, and d) GFDL CM3. Stippling indicates that the 21st and 20th centuries are statistically indistinguishable at the 90% level.

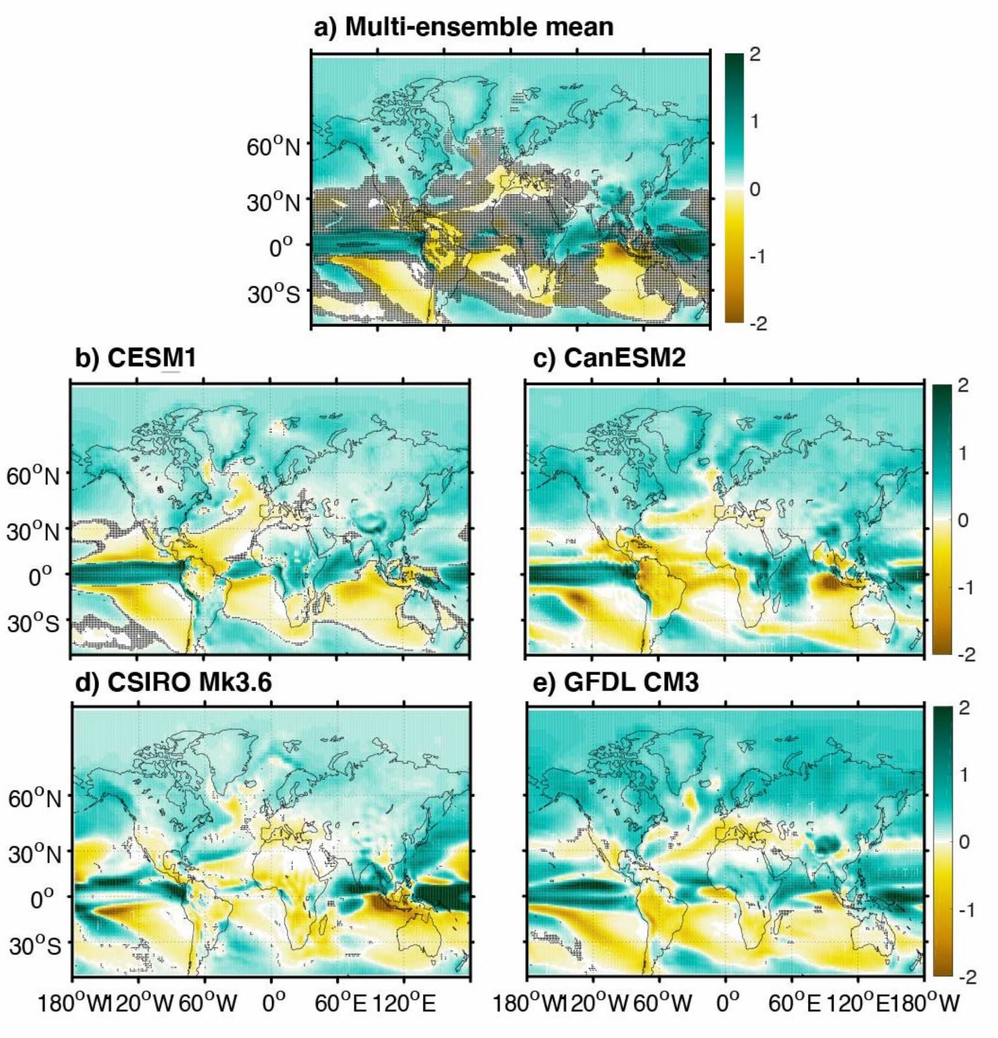

Figure S6: Precipitation difference (mm/day): 21st c - 20th c. a) CESM1, b) CanESM2, c) CSIRO Mk3.6, and d) GFDL CM3. Stippling indicates that the 21st and 20th centuries are statistically indistinguishable at the 90% level.

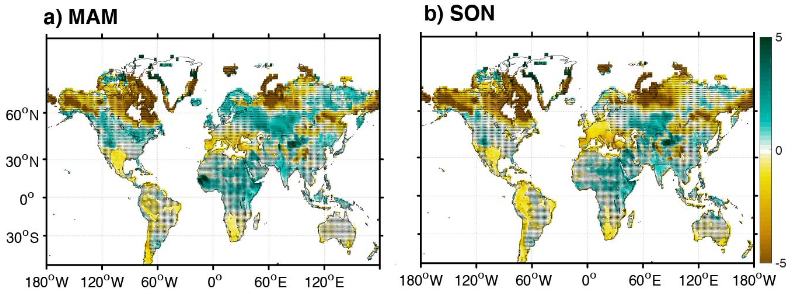

Figure S7: Differences in total column soil moisture in a) MAM and b) SON, averaged across all model ensembles. DIferences are computed between the 2040-2080 and 1950-2005 periods, and are expressed in units of standard deviation normalized to the pre-industrial control simulation as in the main text.

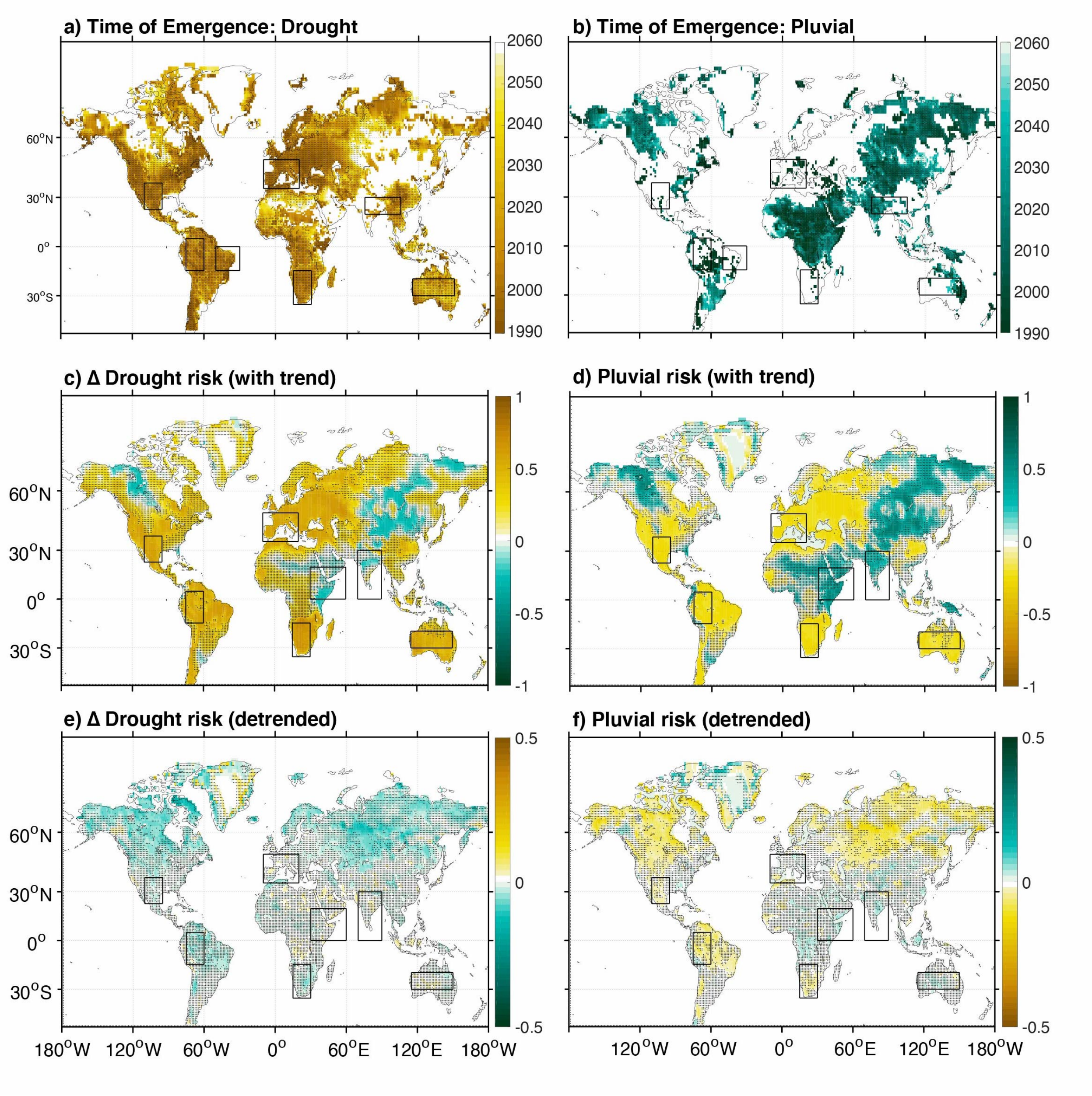

Figure S8: Time of emergence maps derived using surface soil moisture. Threshold for megadrought/megapluvial is -0.5/+0.5σ, and reference period 1960-1990 as in the main text. Only values where 2 or more ensembles agree on the emergence of the signal prior to 2080 are plotted; locations where 3 or more ensembles agree on emergence are stippled.

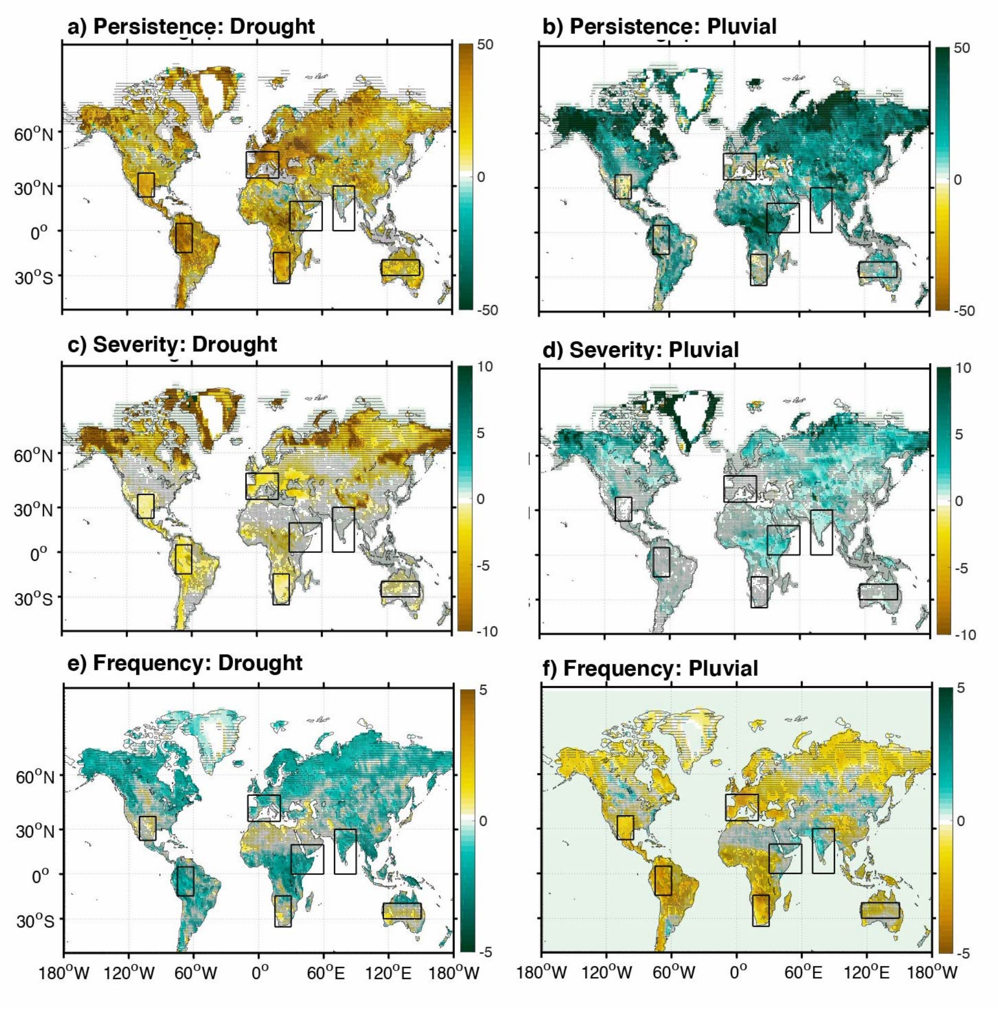

Figure S9: Changes to the properties of megadrought/pluvial events, defined using column soil moisture.

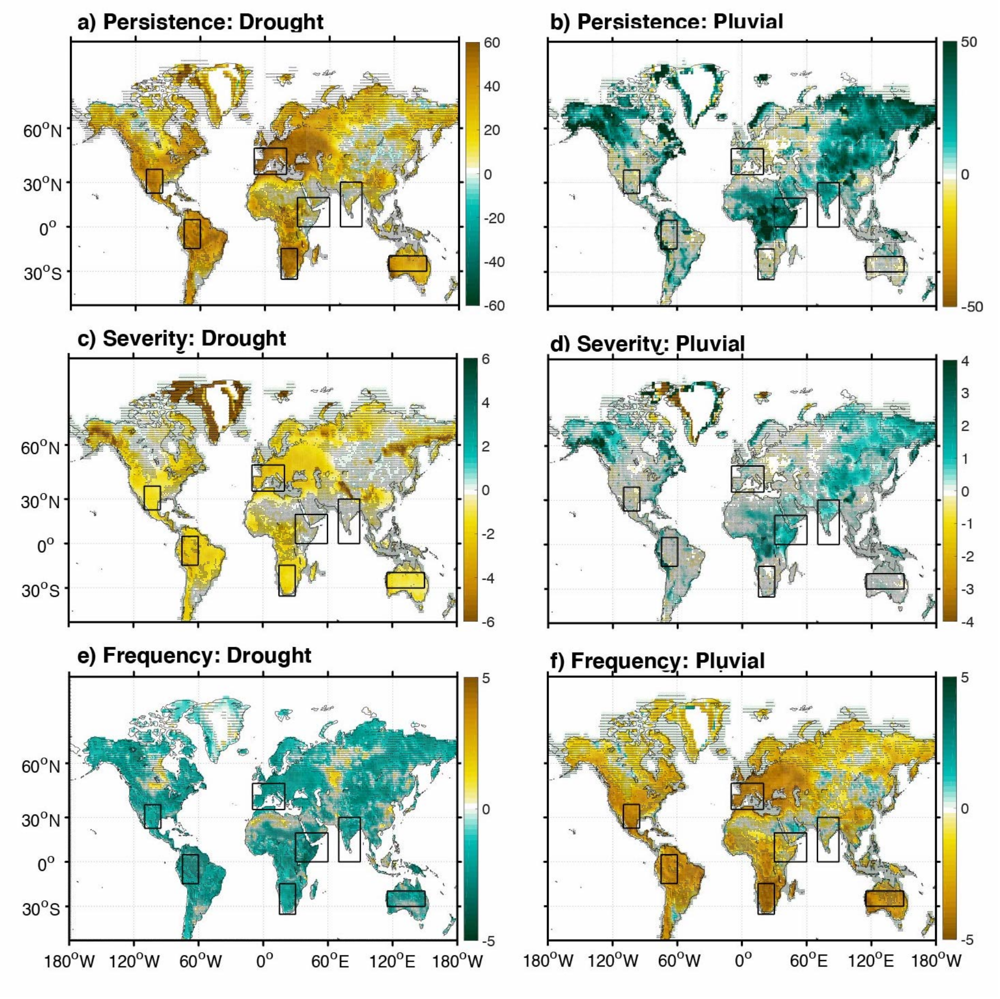

Figure S10: Same as Figure S9, for surface soil moisture.

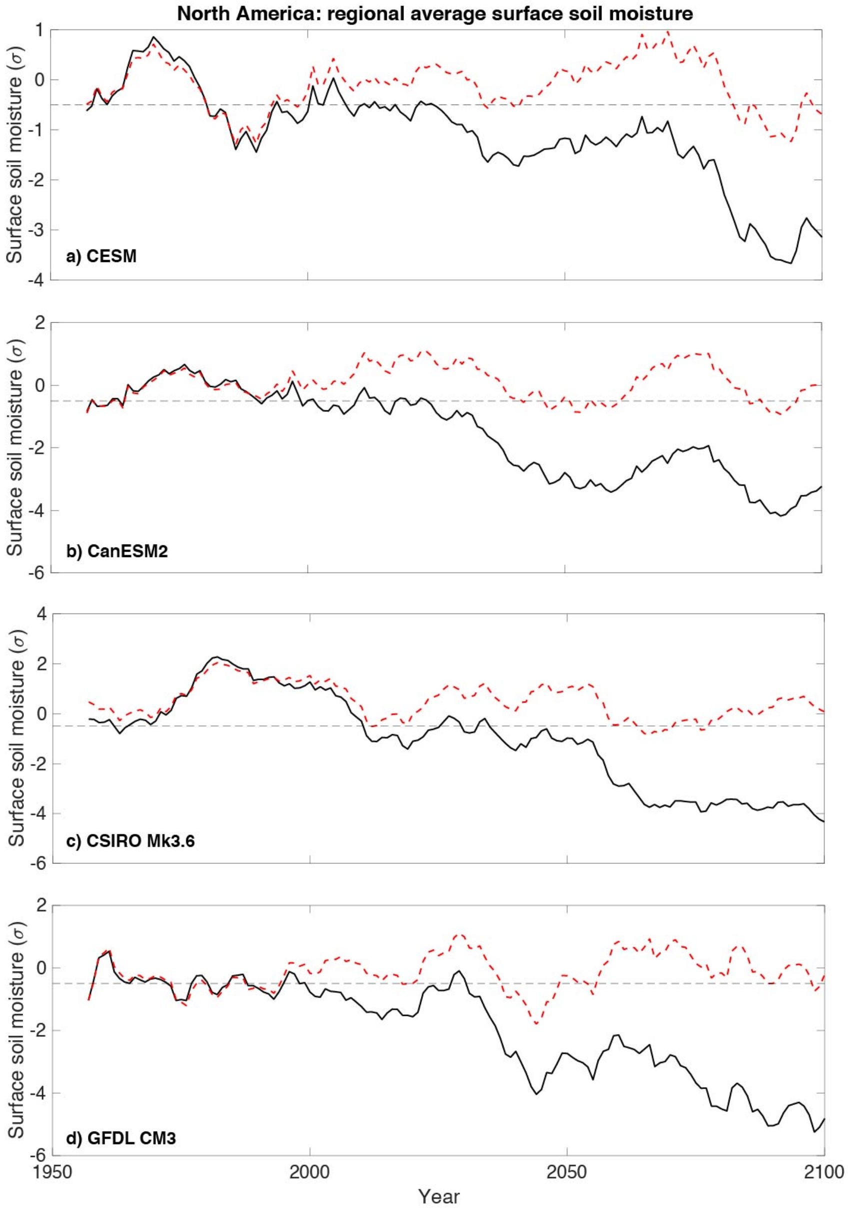

Figure S11: Example time series of regionally averaged soil moisture in the North America region, for a single arbitrarily selected member of each ensemble. Black curve indicates standardized soil moisture including the background trend; red dashed line shows the detrended, standardized soil moisture. Megadrought threshold is shown as the black dashed line.

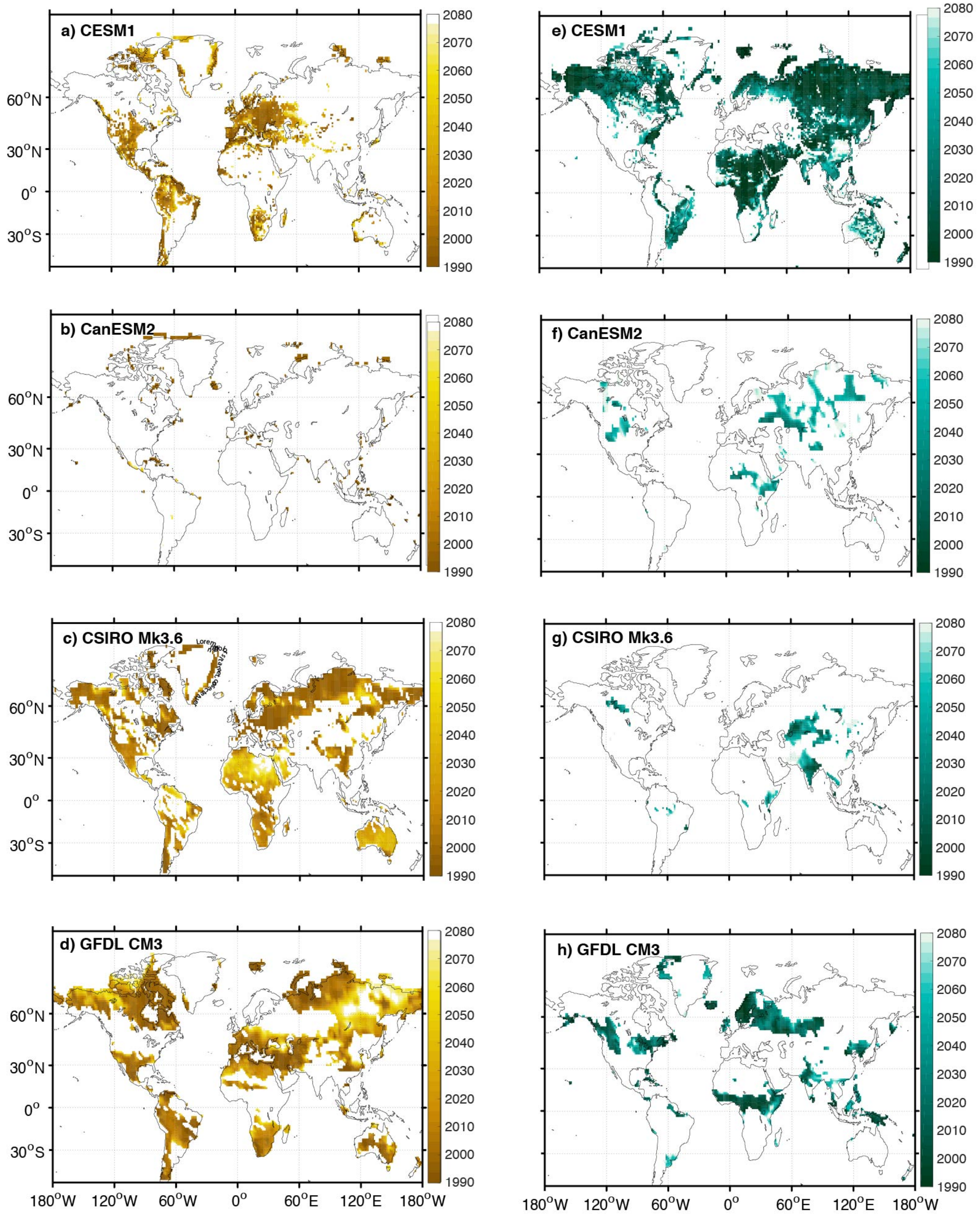

Figure S12: Time of emergence maps for individual model ensembles. a) CESM1, b) CanESM2, c) CSIRO Mk3.6, and d) GFDL CM3. Threshold for megadrought is 0.5σ, and reference period 1960-1990 as in the main text. Total column soil moisture is used in all cases.

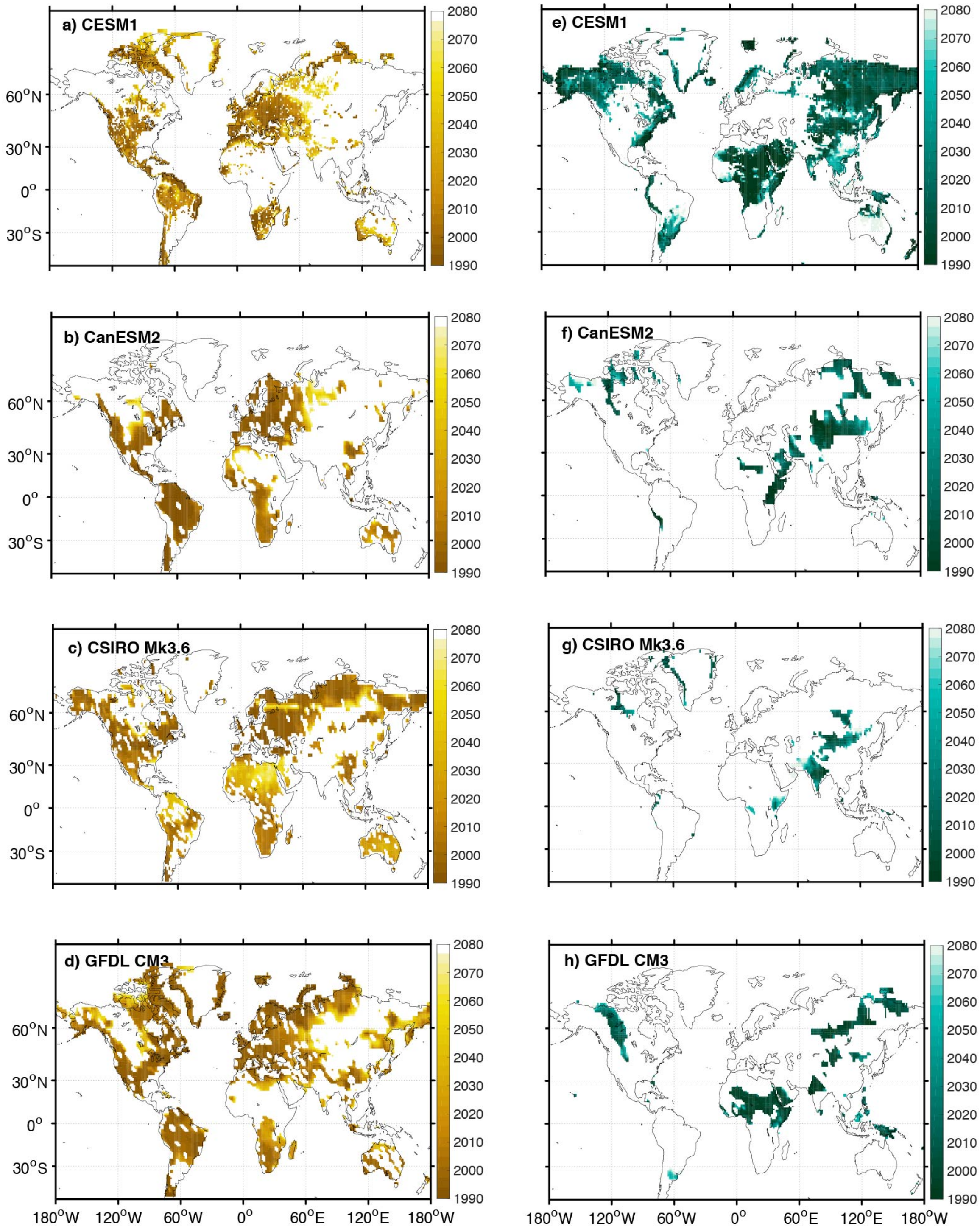

Figure S13: Same as Figure S12, using surface soil moisture.

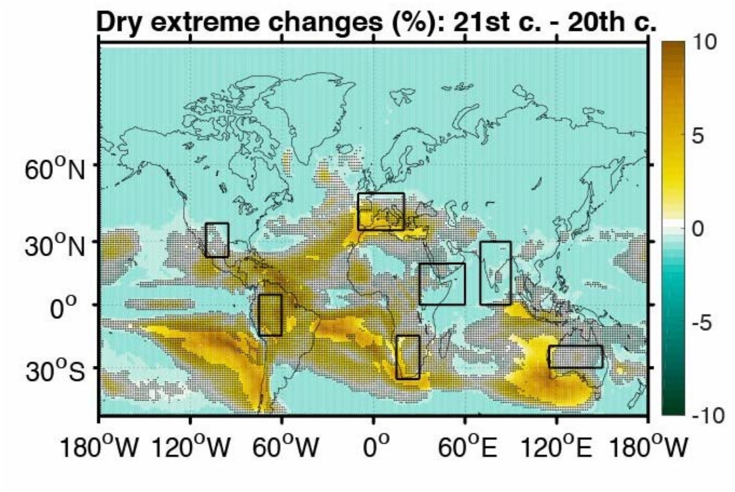

Figure S14: Changes to the occurrence frequency of dry extremes between the 21st and 20th centuries, averaged across all ensembles. Stippling indicates locations where 2 or fewer large ensembles agree on the sign of 21st vs. 20th century changes.

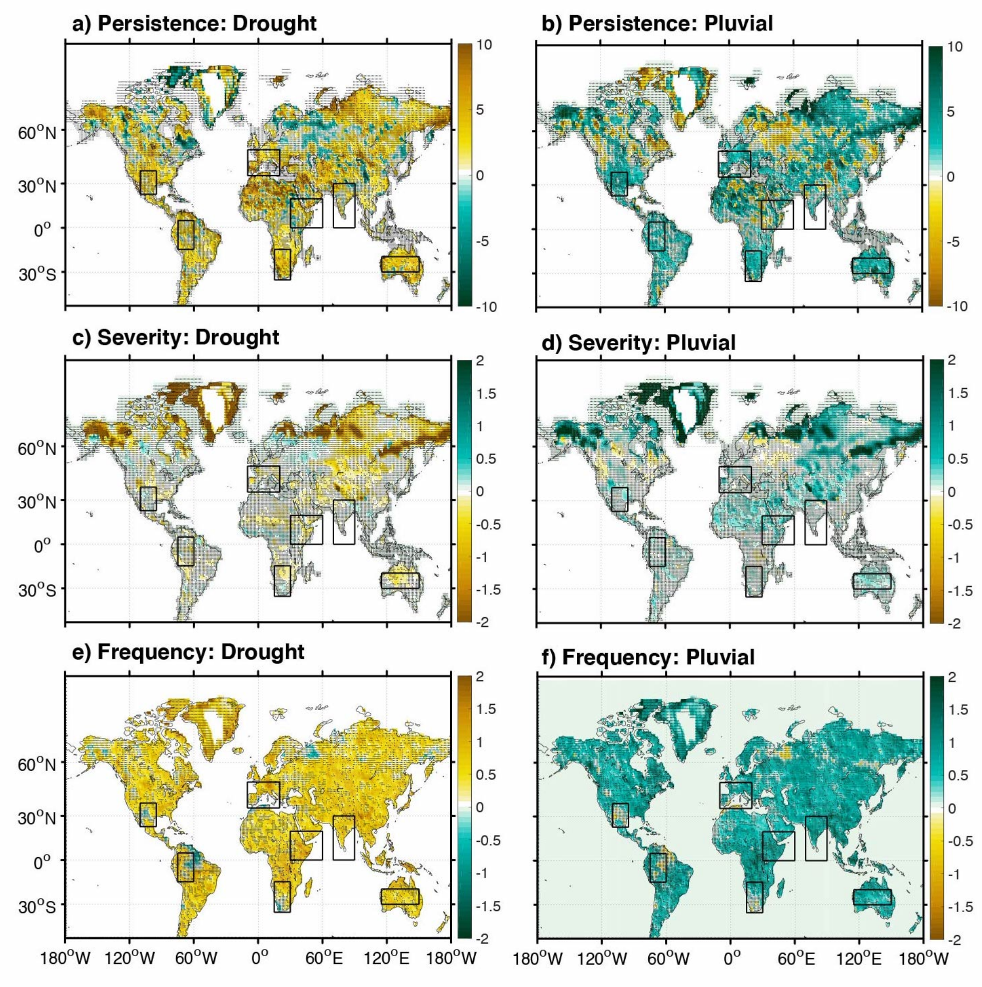

Figure S15: Changes to the properties of detrended megadrought/pluvial events, defined using column soil moisture. a,b: Persistence (years); c,d: Severity (σ); e,f: Frequency (events/century).

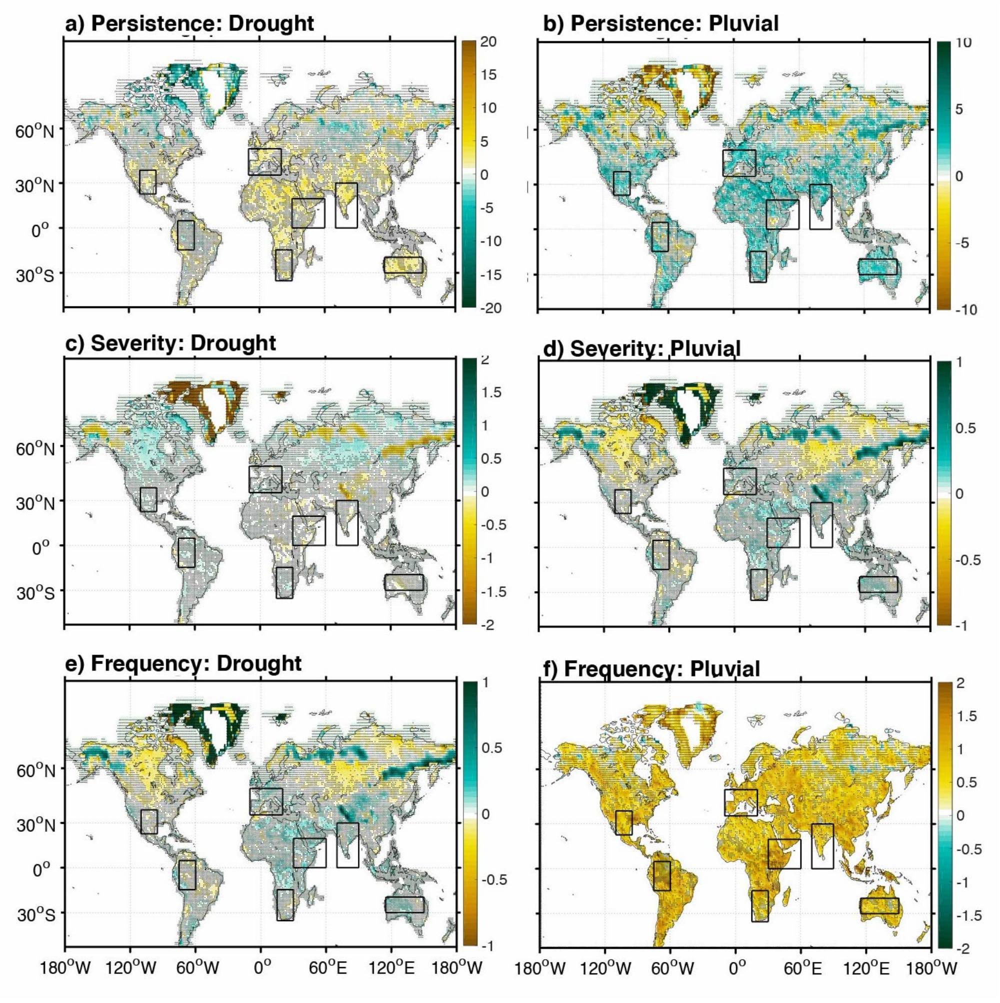

Figure S16: Same as Figure S15, using surface soil moisture.

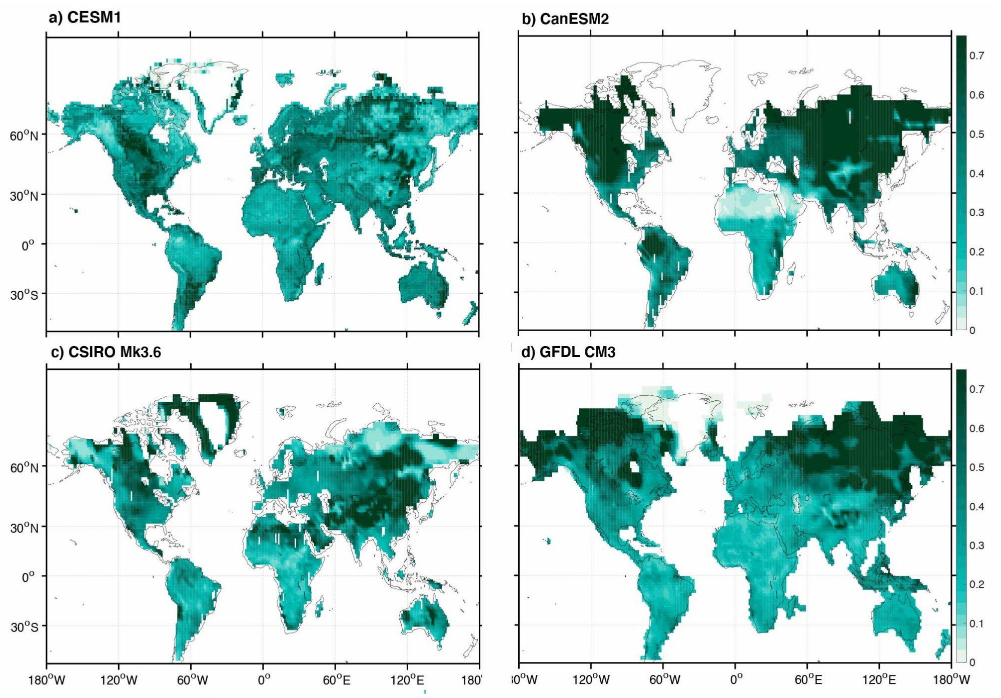

Figure S17: Variance of surface soil moisture (kg m^{-3}) in the pre-industrial control simulations run with: a) CESM1, b) CanESM2, c) CSIRO Mk3.6, and d) GFDL CM3.

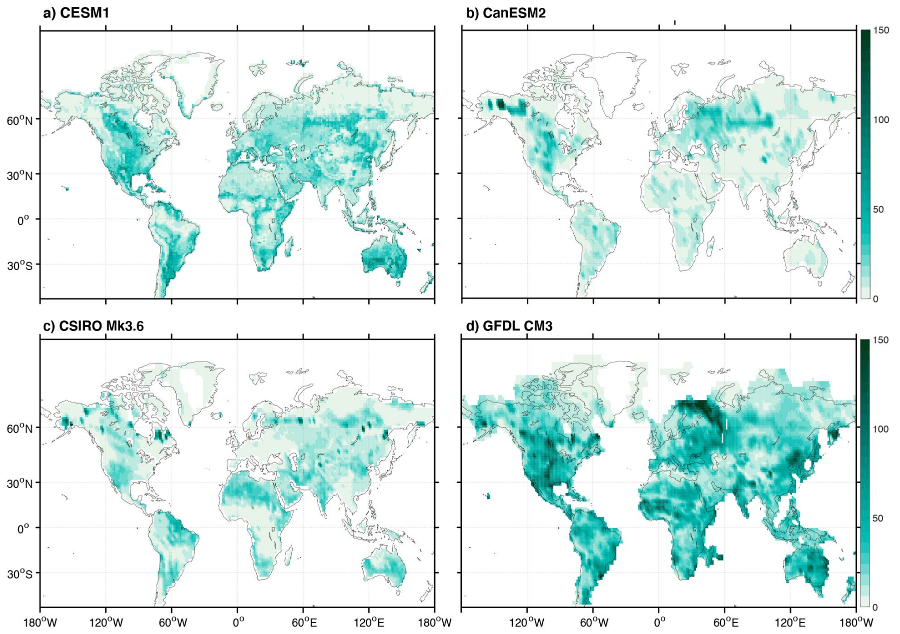

Figure S18: Variance of total column soil moisture (kg m^{-3}) in the pre-industrial control simulations run with: a) CESM1, b) CanESM2, c) CSIRO Mk3.6, and d) GFDL CM3.

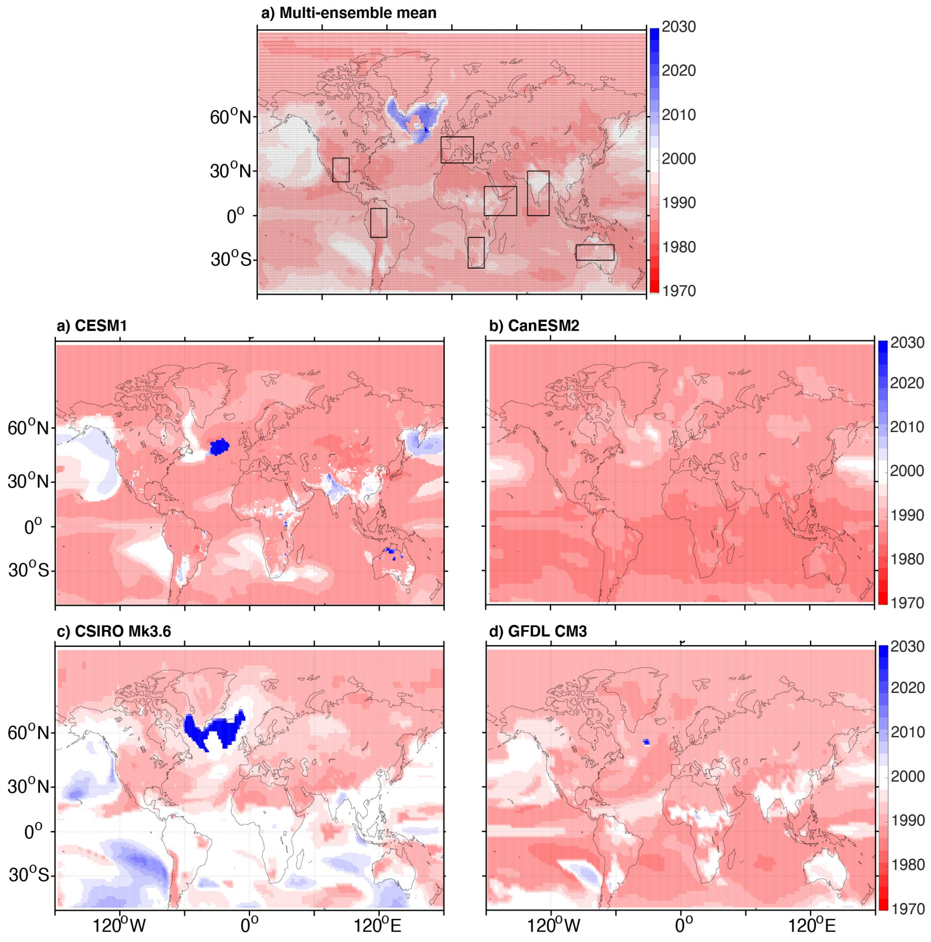

Figure S19: Time-of-emergence calculations for surface temperature: a) multi-ensemble mean; b)-e) individual model ensembles. Stippling in panel a) indicates that 3 or more model ensembles agree that emergence has occurred.

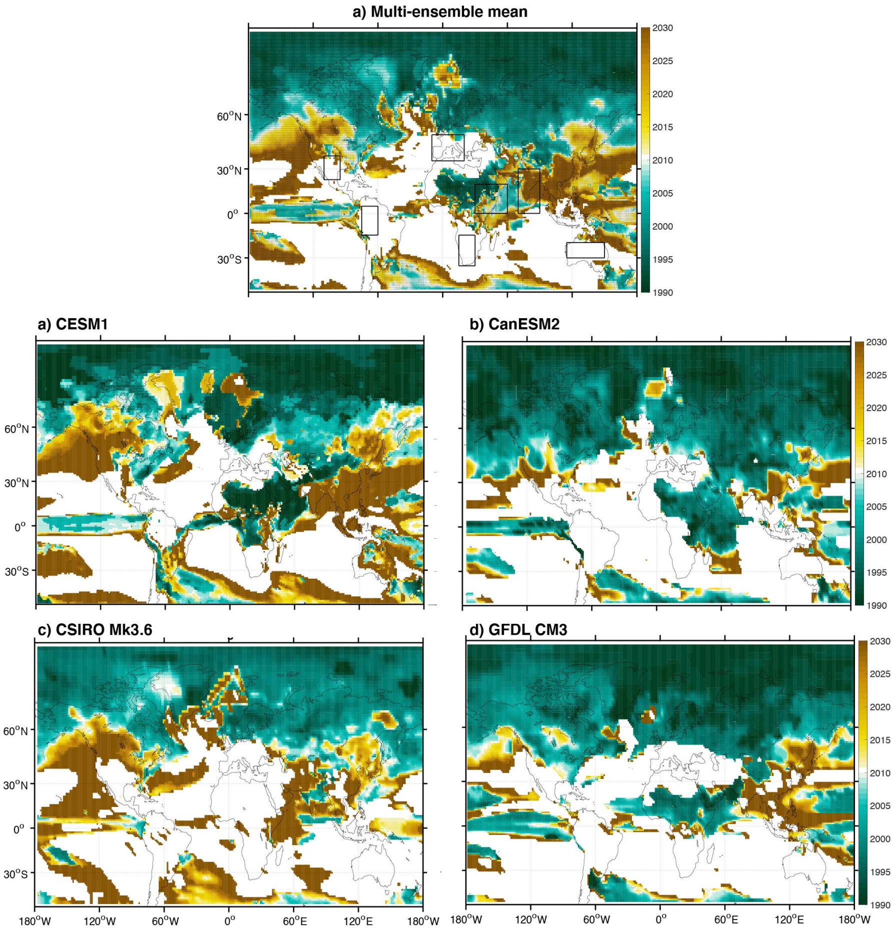

Figure S20: Same as Figure S19, for precipitation.

References

Gordon, H., L. Rotstayn, J. McGregor, M. Dix, E. Kowalczyk, S. O?Farrell, L. Waterman, A. Hirst, S. Wilson, M. Collier, et al., The csiro mk3 climate system model, 2002.

Gordon, H. B., S. O'Farrell, M. Collier, M. Dix, L. Rotstayn, E. Kowalczyk, T. Hirst, and I. Watterson, *The CSIRO Mk3. 5 climate model*, vol. 74, CSIRO and Bureau of Meteorology, 2010.

Jeffrey, S., L. Rotstayn, M. Collier, S. Dravitzki, C. Hamalainen, C. Moeseneder, K. Wong, and J. Syktus, Australia?s cmip5 submission using the csiro-mk3. 6 model, *Aust. Meteor. Oceanogr. J*, *63*(1), 1–14, 2013.

Lawrence, D. M., K. W. Oleson, M. G. Flanner, P. E. Thornton, S. C. Swenson, P. J. Lawrence, X. Zeng, Z.-L. Yang, S. Levis, K. Sakaguchi, et al., Parameterization improvements and functional and structural advances in version 4 of the community land model, *Journal of Advances in Modeling Earth Systems*, *3*(1), 2011.

Milly, P. C., S. L. Malyshev, E. Shevliakova, K. A. Dunne, K. L. Findell, T. Gleeson, Z. Liang, P. Phillipps, R. J. Stouffer, and S. Swenson, An enhanced model of land water and energy for global hydrologic and earth-system studies, *Journal of Hydrometeorology*, *15*(5), 1739–1761, 2014.

Verseghy, D. L., Class?a canadian land surface scheme for gcms. i. soil model, *International Journal of Climatology*, *11*(2), 111–133, 1991.
